# Guyons canal syndrome due to accessory palmaris longus muscle: aetiological classification: a case report

**DOI:** 10.1186/1757-1626-2-9146

**Published:** 2009-12-04

**Authors:** Ramavath Ashok Lal, Sakamuri Raj

**Affiliations:** 1 Department of Orthopaedics, Ysbyty Gwynedd, Bangor, Penrhosgarnedd, North West Wales, LL57 2PW, UK

## Abstract

**Introduction:**

Accessory muscles and anatomic variations are well described at the Guyon's canal. Though this case report is similar to variants published in previous reports, it differs from the rest due to rapidity of worsening of symptoms in few months following use of cane.

**Case presentation:**

We report a case of 69 year old man with ulnar nerve compression at Guyon's canal by accessory palmaris longus arose from distal third palmaris longus and from deep fascia of forearm. The hypertrophied muscular portion of accessory palmaris longus crosses over the ulnar nerve and artery at Guyon's canal becomes tendinous before merging with the hypothenar muscle.

**Conclusion:**

With co-existing anatomical variants, pressure in Guyon's canal might rapidly increase, might be causative factor and cause compression of deep branch of ulnar nerve following frequent dorsiflexion of wrist like in our case. Following division of accessory palmaris longus symptoms rapidly improved. In this article we discuss the aetiological classification, diagnostic criteria and treatment based on available evidence.

## Introduction

Accessory muscles are the most frequently described anatomical variations at Guyon's canal 22.4% in cadaver specimens and bilateral in 46.2% (Dodd's et al., 1990) [1]. Compression of deep branch of ulnar nerve is mainly due to intrinsic or extrinsic factors (Inapathy 2008). The most common anatomical accessory muscle variation were accessory abductor digiti minimi with incidence of 61.5% (Dodds et al., 1990) and accessory palmaris longus with incidence of 8.6%.

This case report differs from other published in the literature in rapidly progression of symptoms following use of cane for to support his new hip replacement. This compression of deep branch of ulnar nerve is confirmed by electro diagnostic studies with predominant compression at Guyon's canal.

## Case presentation

A 69-year-old British Caucasian man presented with pain along hypothenar aspect and severe paraesthesia of six months duration in the area of ring and little finger of left non dominant wrist. The symptoms are aggravated rapidly with progressive weakness following use of cane. This cane was prescribed to support his new revision hip. On further examination in out patient department, revealed hypothenar atrophy and wasting of interosseous muscles, hypoesthesia and paraesthesia over ring and little finger. The left hand grip was weak. Tinel sign positive at wrist. The symptoms were reproduced by Phalen's test.

The electrodiagnostic studies revealed, slowing of ulnar motor and sensory mainly at the wrist. The electro diagnostic study of median nerve was normal. A diagnosis of ulnar nerve entrapment in Guyon's canal made and subsequently patient underwent decompression with good recovery.

**Figure 1:**
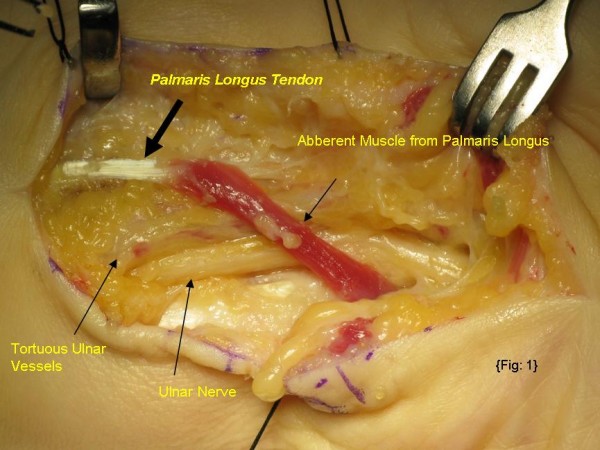
**Aberrant palmaris longus muscle compressing ulnar nerve and vessels at Guyon's canal**. Note: tortuous ulnar vessels.

**Figure 2:**
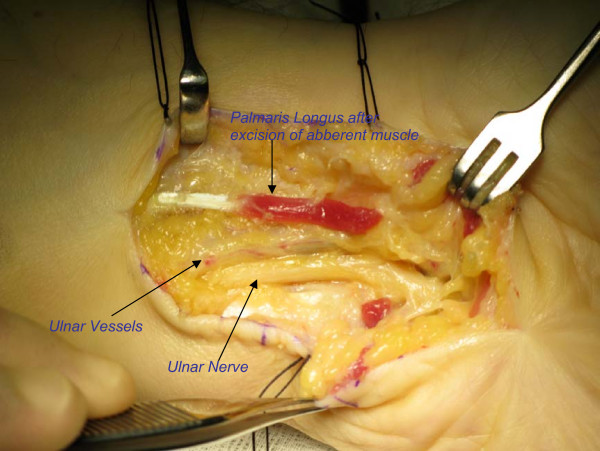
**Release of compression over ulnar nerve and vessel at Guyon's canal**. Note: excised ends of aberrant palmaris longus muscle.

At operation, following surgical exploration of Guyon's canal, revealed accessory palmaris longus muscle originating from the palmaris longus tendon and few muscle fibres from deep fascia of forearm passing through Guyon's canal over the ulnar nerve and vessels and merging with hypothenar muscles (Figure 1). We also noticed tortuous ulnar vessels proximal to Guyon's canal. This hypertrophied muscle was compressing common ulnar nerve trunk and ulnar vessel. The muscle was divided widely and decompressed ulnar nerve and ulnar vessel (Figure 2). The ulnar nerve was explored proximally three to four inches in to distal fore arm and did not find any other sites of compression. There was no constriction at pisohammate hiatus. After hemostasis and skin closure, a soft bandage applied and no post operative complications detected. Post operatively patient under went hand physio, and free of compression symptoms following review at OPD in two and six weeks.

## Discussion

Following the review of literature, [2] based on aetiology, the causes of ulnar tunnel syndrome broadly categorized in to primary and secondary.

The primary is idiopathic with symptoms of Guyon's canal syndrome with no identifiable lesion, in a study done by Murata et al 2004 found 45% of cases were idiopathic. The secondary causes can be further divided in to traumatic, inflammatory, structural, vascular (Ozdemir 2007), neuropathic and occupational. Following fractures to hook of hammate [3,4] fractures of ring and little finger metacarpal bases there were reported evidence of compression of ulnar nerve at the wrist. Inflammatory conditions like rheumatoid arthritis, rheumatoid synovitis can also cause compression of ulnar nerve [5].

Structural causes can be further divided in to tumorous and nontumorous. The tumorous conditions causing compression over deep branch of ulnar nerve are ganglion [6], lipoma [7], and giant cell tumour (Hayes JBJS 1978).

Anomalies at Guyon's canal are common 22.4% and bilateral (46.2%) in cadaver specimens [1]. The radiographic study by Ziess et al1992 incidence of accessory muscle is 16.2%. Non tumorous conditions are anomalous muscles like unusual thickening of distal edge of palmaris brevis (Mackinon 1989), thickening of proximal edge of volar carpal ligament or fibrous origin of flexor digiti minimi muscle [8], complete reversal of palmaris longus so that tendon arises from the medial epicondyle and muscle belly attaches to flexor retinaculum at the wrist [9], accessory palmaris longus muscle arise from palmaris longus tendon insert distally in to pisiform bone and in to the hypothenar muscle traversing through the Guyon's canal (Thomas JBJS 1958), thickening of roof of Guyon's canal due to origin of anomalous muscle from the flexor carpi ulnaris inserting in to the volar carpal ligament [10], High origin of flexor digiti minimi from palmaris longus, with double origin reported (Jeffrey JBJS1971), duplication of hypothenar muscles simulating a hand tumour described (Lipscomb JBJS 1960), the most common muscle variant at Guyon's canal is abductor digiti minimi (61.5%) which passes through Guyon's canal [1], in one reported case its anomalous variant arose from transverse carpal ligament instead of pisiform bone [11].

The vascular causes of Guyon's canal syndrome are ulnar artery aneurysm (Yoshii 1999) or ulnar artery thrombosis [12], pain in hypothenar region followed by continuous tremors of ring and little finger is due to distal ulnar neuropathy relieved by Guyon's canal decompression [13].

The occupational or social causes are mostly contributory due to over use like in carpenters and in our case, resulting in compression of deep branch of ulnar nerve worsened following use of cane with or with out anatomical variation.

The diagnosis of Guyon's canal syndrome made by history, clinical examination and electromyography studies. One can clearly differentiate ulnar nerve compression at wrist or above elbow, by eliciting weakness of flexor carpi ulnaris and flexor digitorum profundus to ring and little finger metacarpal and sensory changes to ulnar side dorsum hand. Detailed electro diagnostic studies help in diagnosis.

## Conclusion

With above check list in mind and careful clinical examination one can diagnose Guyon's canal syndrome. Though anomalies are frequent at Guyon's canal, over use with frequent dorsi flexion position might have aggravated symptoms, early diagnosis followed by exploration and decompression will relieve symptoms like described in our case.

## Consent

Written informed consent was obtained from the patient for publication of this case report and accompanying images. A copy of the written consent is available for review by the Editor-in-Chief of this journal.

## Competing interests

The authors declare that they have no competing interests.

## Authors' contributions

RA - main author, wrote the paper, performed the literature search and discussion.

SR - aided in editing and senior author of this article.

## References

[B1] Dodds GA Hale D Jackson WT Incidence of anatomic variants in Guyon's canal J Hand Surg Am 1990 15-A 352 355 10.1016/0363-5023(90)90122-8 2324469

[B2] Bozkurt MC Tağil SM Ozçakar L Ersoy M Tekdemir I Anatomical variations as potential risk factors for ulnar tunnel syndrome: a cadavaric study Clin Anat 2005 18 274 280 10.1002/ca.20107 15832354

[B3] Vance RM Gelberman RH Acute ulnar neuropathy with fractures at the wrist J Bone Joint Surg Am 1978 60 962 965 701345

[B4] Gore DDR Carpometacarpal dislocation producing compression of the deep branch of ulnar nerve J Bone Joint Surg Br 1971 53 1387 1390 4329822

[B5] Taylor AR Ulnar nerve compression at the wrist in rheumatoid arthritis J Bone Joint Surg Br 1974 56 142 143 4818842

[B6] Inaparthy PK Anwar F Botchu R Jahnich H Katchburian MV Compression of the deep branch of the ulnar nerve in Guyon's canal by a ganglion: two cases Arch Orthop Trauma Surg 2008 128 7 641 643 10.1007/s00402-008-0636-4 18509691

[B7] McFarland GB Hoffer MM Paralysis of intrinsic muscle of hand secondary to Lipoma in Guyon's canal J Bone Joint Surg Am 1971 53 375 376 5546713

[B8] Uribru IJF Morchio FJ Marin JC Compression syndrome of the deep motor branch of the ulnar nerve (pisohammate hiatus syndrome) J Bone Joint Surg Am 1976 58 145 147 1249106

[B9] Spinner M Freundlich BD An important variation of the palmaris longus Bull Hospital Jt Dis 1967 28 126 130 5583648

[B10] Spinner M Spinner M From The ulnar nerve Disorders of Major Peripheral Nerves 1978 2 Philadelphia: WB Saunders 245 266

[B11] Netscher D Victor C Ulnar nerve compression at the wrist secondary to anomalous muscles: A patient with variant of abductor digiti minimi Ann Plast Surg 1997 39 6 647 651 10.1097/00000637-199712000-00017 9418928

[B12] Smith RJ Ulnar nerve compression secondary to ulnar artery false aneurysm at Guyon's canal (letter) J Hand Surgery 1982 7 631 632 10.1016/s0363-5023(82)80121-4 7175141

[B13] Streib EW Distal ulnar neuropathy as a cause of finger tremor: a case report Neurology 1990 40 1 153 154 2153273 10.1212/wnl.40.1.153

